# Long-Term Outcomes of a Therapist-Supported, Smartphone-Based Intervention for Elevated Symptoms of Depression and Anxiety: Quasiexperimental, Pre-Postintervention Study

**DOI:** 10.2196/14284

**Published:** 2019-08-26

**Authors:** Marcos Economides, Kristian Ranta, Albert Nazander, Outi Hilgert, Philippe R Goldin, Anu Raevuori, Valerie Forman-Hoffman

**Affiliations:** 1 Meru Health Inc Palo Alto, CA United States; 2 Betty Irene Moore School of Nursing University of California Davis Sacramento, CA United States; 3 Clinicum, Department of Public Health University of Helsinki Helsinki Finland; 4 Department of Adolescent Psychiatry Helsinki University Central Hospital Helsinki Finland

**Keywords:** digital health, depression, anxiety, mindfulness, CBT, online intervention, smartphone intervention

## Abstract

**Background:**

Depression is one of the most common mental health disorders and severely impacts one’s physical, psychological, and social functioning. To address access barriers to care, we developed Ascend—a smartphone-delivered, therapist-supported, 8-week intervention based on several evidence-based psychological treatments for depression and anxiety. A previous feasibility study with 102 adults with elevated depression reported that Ascend is associated with a postintervention reduction in depression symptoms.

**Objective:**

We aimed to examine whether Ascend is associated with a reduction in symptoms of anxiety, and importantly, whether reductions in symptoms of depression and anxiety are maintained up to 12-months postintervention.

**Methods:**

We assessed whether the previously reported, end-of-treatment improvements seen in the 102 adults with elevated symptoms of depression extended up to 12 months posttreatment for depression symptoms (measured by the Patient Health Questionnaire-9 [PHQ-9]) and up to 6 months posttreatment for anxiety symptoms (added to the intervention later and measured using the Generalized Anxiety Disorder-7 [GAD-7] scale). We used linear mixed effects models with Tukey contrasts to compare time points and reported intention-to-treat statistics with a sensitivity analysis.

**Results:**

The intervention was associated with reductions in symptoms of depression that were maintained 12 months after the program (6.67-point reduction in PHQ-9 score, 95% CI 5.59-7.75; *P*<.001; Hedges g=1.14, 95% CI 0.78-1.49). A total of 60% of the participants with PHQ-9 scores above the cutoff for major depression at baseline (PHQ≥10) reported clinically significant improvement at the 12-month follow-up (at least 50% reduction in PHQ-9 score and postprogram score <10). Participants also reported reductions in symptoms of anxiety that were maintained for at least 6 months after the program (4.26-point reduction in GAD-7 score, 95% CI 3.14-5.38; *P*<.001; Hedges g=0.91, 95% CI 0.54-1.28).

**Conclusions:**

There is limited evidence on whether outcomes associated with smartphone-based interventions for common mental health problems are maintained posttreatment. Participants who enrolled in Ascend experienced clinically significant reductions in symptoms of depression and anxiety that were maintained for up to 1 year and 6 months after the intervention, respectively. Future randomized trials are warranted to test Ascend as a scalable solution to the treatment of depression and anxiety.

## Introduction

Depression is a common mental health disorder and one of the leading causes of disease burden and disability worldwide [1-3]. Individuals with depression have a reduced capacity to work and function in daily life, causing major economic and societal costs [4-6]. By 2030, depression is predicted to pose the largest burden of disease in high-income countries, surpassing heart disease, dementia, and alcohol-related disorders [1].

Although there are several effective pharmacological [7] and psychological [8,9] treatments for depression, less than half of all individuals who require treatment actually receive it [10,11]. Barriers to treatment include financial and time constraints, long wait periods, a shortage of trained professionals, and fear of stigmatization [12,13]. When treated with antidepressants, an estimated 30%-50% of patients do not experience significant symptom reduction [14], up to 80% of patients report at least one mild-to-severe side effect [15], and few patients maintain a state of long-term remission [16]. Estimates suggest that up to 70% of individuals with depressive disorders have a comorbid anxiety disorder, which renders treatment even more challenging [17]. Further, up to 75% of patients referred to in-person psychotherapy either do not enter treatment or discontinue treatment prematurely [18,19]. Thus, there is an urgent need for novel, evidence-based treatments for depression and anxiety to overcome these barriers.

Recently, digital interventions delivered via smartphone apps have been developed as a means to address this need [20]. Smartphone ownership has seen rapid worldwide growth [21], and survey data suggest that smartphone-based interventions may be preferred over other online formats by health care consumers [22,23]. Smartphone-based interventions offer several advantages over traditional treatment modalities including large-scale accessibility and scalability; low costs; patient anonymity and privacy; standardized content that is less dependent on therapist skills; flexible usage at a self-determined time and pace, which is thought to enhance self-efficacy [24]; monitoring of activity, symptoms, and progression in real time; provision of personalized feedback, motivational support, and targeted care; and potential to improve adherence to treatment [25]. Preliminary evidence suggests that smartphone-based interventions are a promising means to treat depression and anxiety, with recent meta-analyses reporting small-to-moderate reductions in clinical symptoms across 27 studies [26,27].

Despite these promising results, several important questions regarding the design, efficacy, and implementation of smartphone-based interventions for common mental health disorders remain unanswered. First, few authors report on the outcomes of commercially developed smartphone-based interventions for depression and anxiety, making it difficult to evaluate their utility [25,28]. Second, despite being generally effective for the short-term treatment of symptoms of depression and anxiety, there is a dearth of evidence regarding how long the beneficial effects of smartphone-based interventions and other online interventions are maintained posttreatment [29]. This is important because an estimated 50% or more of patients with major depression or generalized anxiety disorder will relapse within 6 to 12 months after the end of an initially effective treatment [30-32]. Thus, investigation of the long-term outcomes associated with smartphone-based interventions for depression and anxiety is an important step toward understanding their true real-world effectiveness. Lastly, despite an increased interest in transdiagnostic interventions that target depression and anxiety concurrently [33], most smartphone-based interventions for mental health are disorder-specific, and it remains unknown whether smartphone-based interventions can address symptoms of depression and anxiety when they are comorbid.

We recently evaluated the feasibility of the Meru Health *Ascend* intervention, a novel, 8-week smartphone-based intervention for elevated symptoms of depression and anxiety, assisted by a remote therapist [34]. The intervention was found to be feasible and was associated with a postintervention reduction in depression. In this study, we extend these findings by investigating whether the previously reported postintervention reductions in depression are maintained at a 1-year follow-up, whether *Ascend* is associated with reductions in comorbid symptoms of anxiety, and whether any postintervention reductions in symptoms of anxiety are maintained at a 6-month follow-up. Thus, this study aims to lend important insights into the real-world, long-term impact of smartphone-based interventions for the treatment of common mental health problems. We hypothesized that postintervention reductions in symptoms of depression would be maintained at the 1-year follow-up and that Ascend would be associated with reductions in the symptoms of anxiety, which would persist at the 6-month follow-up. Lastly, as a secondary objective, we aimed to examine whether participant demographics or intervention engagement were predictive of symptom change or attrition at follow-up.

## Methods

### Research Design

We used a quasiexperimental research design that included a single-arm, pre- and postintervention assessment of outcomes. Symptoms of depression were measured before the intervention (“baseline”); at the end of the 8-week intervention; and at 1, 3, 6, and 12 months postintervention. Symptoms of anxiety were measured at baseline; week 4 of the intervention; the end of the 8-week intervention; and 1, 3, and 6 months postintervention.

### Participants

This study included adult patients treated at the Meru Health Online Clinic, a national remote health care provider that currently operates in the United States and Finland. The clinic has had a rolling enrolment since March 2017. At the time of this study, 197 enrollees had passed the 6-month postintervention outcome window (recruited between March 2017 and June 2018), and 102 passed the 12-month postintervention outcome window (recruited between March and December 2017). Our primary analysis includes the latter group, although we also report the Patient Health Questionnaire (PHQ-9) results from the former group with 6-month postintervention outcomes only. The *Ascend* intervention was primarily intended to treat symptoms of depression; however, many individuals with depression also have comorbid symptoms of anxiety [17]. Thus, to evaluate whether *Ascend* is also associated with reductions in symptoms of anxiety, Generalized Anxiety Disorder-7 (GAD-7) scale measures were added to the intervention assessment in December 2017. At the time of this study, 102 of the original 197 enrollees with 6-month postintervention PHQ-9 outcomes also had GAD-7 (anxiety) data (recruited between January and June 2018), while 12-month postintervention anxiety data were not yet available for any participants. Note that the 102 participants with 6-month postintervention GAD-7 data are different from the 102 participants with 12-month postintervention PHQ-9 data.

Participants were recruited via online Facebook advertisements that sought participants for a smartphone-based intervention for depression that included self-guided smartphone-delivered content, private access to a therapist via messaging, and an anonymous group-chat feature with other participants. Prior to the intervention, participants were given free access to the app and trained on how to use the group-chat feature and communicate with their assigned therapist. Participant demographics (age, gender, and antidepressant medication status) were acquired at the start of the intervention via an intake questionnaire administered online. Since medication status was introduced in July 2017, these data are absent for the first 33 participants. Outcome measures were administered via the app for all within-intervention time points (including immediately postintervention) and via email for all follow-up time points.

For inclusion, participants had to provide informed consent via the Meru Health app, own a smartphone, have at least mild symptoms of depression (a score≥5 on the PHQ-9 at baseline), and acknowledge/demonstrate the ability to commit to a minimum of 20 minutes of practice per day for 6 days per week across the 8-week intervention (as judged by both the participant and their assigned therapist). Exclusion criteria included a previous suicide attempt, severe active suicidal ideation with a specific plan, severe self-harm, active substance abuse, and a history of psychosis. Inclusion/exclusion criteria were assessed prior to enrolment via phone interviews between study participants and intervention therapists, as per the standard treatment procedure at the Meru Health Online Clinic. Participants were not compensated for their time but could participate in the intervention for free. All participants provided informed consent for their anonymized data to be used for research purposes prior to engaging with the intervention. All procedures were reviewed by the Pearl Institutional Review Board, which granted exemption for analyses of previously collected and deidentified data. All procedures performed were in accordance with the 1964 Helsinki declaration and its later amendments or comparable ethical standards.

An a priori sample size calculation was performed for comparing patient-reported outcome measures (PHQ-9 and GAD-7 scores) at baseline and follow-up time points. Using an alpha level of .05, a power of 0.8, and a medium effect size of 0.5, 33 subjects were needed. Thus, this study was sufficiently powered to detect a medium effect, even after accounting for substantial dropout over the course of the 1-year follow-up period.

### Intervention

The Meru Health *Ascend* intervention has been described in detail previously [34]. Briefly, the intervention consists of 8 modules delivered sequentially over an 8-week period, which include content derived from evidence-based practices such as mindfulness-based stress reduction [35], mindfulness-based cognitive therapy [36], cognitive-behavioral therapy [37], and behavioral activation therapy [38]. The content includes text; video; audio-guided mindfulness meditation exercises; infographics that illustrate cognitive-behavioral therapy principles; and journal prompts. Daily content and practices range from 10 to 30 minutes, except for the first day of each week, in which a series of introductory videos extend the content to a maximum of 45 minutes. The Meru Health app can be used on both Android and iOS and is designed to be platform-agnostic and thus equivalent across different operating systems.

A licensed therapist (employed by Meru Health) provides support to participants via messaging (and less frequently, phone calls), throughout the intervention. As part of this support, therapists review practice logs using a provider “dashboard” and electronic medical records (that detail participant engagement and patient-reported outcomes to date) to monitor individual participant progress. Therapists aim to spend approximately 20 minutes (on average) supporting each participant per week of the intervention (including initiating contact at least 2-3 times per week), but are at liberty to adjust the levels of support in accordance with each participant’s individual progress. In addition, participants are free (and encouraged) to contact their allocated therapist when they require additional support. Such two-way interaction is designed to create a system of support that is structured while being tailored to each participant’s personal preference and needs. Further, therapists are instructed to conduct a phone-based assessment for any participants that show signs of mental deterioration during or immediately after the intervention. In case of an emergency, such as severe suicidality, the intervention includes a written security plan, which all participants are required to review with their therapist before engaging with the intervention.

Participants are enrolled in groups of 10-15 individuals that work through the intervention at the same time and can provide anonymous support to one another via a discussion board within the app. Specifically, participants can post anonymous reflections on practices and lessons to the discussion board, to which their therapist can respond freely, and to which other group members can respond with prewritten empathy statements and emoticons. Free cross-talk between participants is not allowed.

In Finland, Meru Health is approved by the Finnish National Supervisory Authority for Welfare and Health (Valvira approval number V/25535/2017) and is compliant with the European Union General Data Protection Regulation. In the United States, Meru Health is compliant with HIPAA (Health Insurance Portability and Accountability Act of 1996) legislation. All protected health information is kept in a HIPAA-compliant electronic medical record, which is housed in cloud-based storage systems hosted by a company named Datica [39]. All data are encrypted in transit, end-to-end, and at rest.

### Measures

#### Patient Health Questionnaire

The PHQ-9 is a 9-item depression scale, derived from the full PHQ and is one of the most widely used instruments to screen for the presence and severity of depression in primary care [40]. Participants rate each item on a Likert scale from 0 (not at all) to 3 (nearly every day), with total scores ranging from 0 to 27. In general, a score of 10 or above suggests the presence of major depression, and scores of 5, 10, 15, and 20 are taken as cut-off points for mild, moderate, moderately severe, and severe depression, respectively. The PHQ-9 has excellent internal consistency (Cronbach α of 0.89 in primary care settings) and excellent test-retest reliability [41]. In their original validation study, Kroenke and colleagues [40] defined a clinically significant improvement in depression as a 50% reduction in the PHQ-9 score combined with a postintervention score of <10 (for participants with baseline scores≥10), and this definition has been further validated in a comparison study [42]. A similar yet more liberal definition for clinically significant change was also proposed by Löwe and colleagues [43] as a PHQ-9 score reduction of ≥5.

#### Generalized Anxiety Questionnaire

The GAD-7 is a 7-item scale used extensively in outpatient and primary care settings to screen for the presence and severity of an anxiety disorder [44]. Participants rate each item on a Likert scale from 0 (not at all) to 3 (nearly every day), with total scores ranging from 0 to 21. In general, a score of 10 or above is suggestive of the presence of anxiety to the extent that further evaluation is warranted, and scores of 5, 10, and 15 are taken as cut-off points for mild, moderate, and severe anxiety, respectively. The GAD-7 has excellent reliability and internal consistency (Cronbach α of 0.89) and has been validated in both the general population and primary care settings [44,45]. A clinically significant improvement in anxiety symptoms has previously been defined as a GAD-7 score reduction of ≥3 [46].

### Statistical Analysis

#### Measures of Engagement

Descriptive statistics were calculated for participant demographics and week-by-week engagement metrics. For each participant, we calculated a measure of intervention engagement, defined as total days in which >3 minutes of app-based meditation was completed. We used a threshold of 3 minutes, as this corresponds to the shortest meditation session available in the intervention. We used logistic regression to explore whether baseline characteristics were predictive of the completion of outcome measures at 6 and 12 months postintervention. Explanatory variables included age, gender, country, intervention engagement (as defined above), and baseline PHQ-9 or GAD-7 scores.

#### Patient-Reported Outcomes

Outcome measures were analyzed using an intention-to-treat analysis in which all participants with outcome measures at baseline were included, regardless of intervention engagement or attrition. We used linear mixed effects models (LMMs) implemented through LME4 [47] and Tukey contrasts to compare between time points (using the “multcomp” package [48]) in the statistical computing software R [computer software] (version 3.5.2. Vienna, Austria: R Foundation for Statistical Computing). LMMs are capable of handling missing data and are considered superior to other ITT approaches such as the last observation carried forward [49]. We modeled a single within-subject factor “time” (as a fixed effect) and a separate baseline for each participant (random-intercept model). Time was modeled as a categorical predictor, and we therefore did not enforce a linear relationship between time and outcome measures. We also ran the model while controlling for participant age, gender, country, total intervention engagement (total active days), and total therapist contact, both with and without two-way interactions with time, which produced equivalent results. We report the contrast estimate, 95% CI of the estimate, and  *P* value.  *P* values<.05 were considered significant. The estimated marginal mean and standard error for each time point was calculated using the “emmeans” package in R.

#### Sensitivity Analysis

Since LMMs rely on data being “missing at random,” an assumption that is difficult to verify in clinical research, we also implemented an LMM-based pattern-mixture model (PMM) for the analysis of PHQ-9 (depression) scores. PMMs are based on a joint modeling of outcomes and missingness and can account for data that is “missing not at random” [50]. We modified the estimated marginal mean (EMM) from the previously described LMM at the 6- and 12-month time points based on specific clinical assumptions about participants with missing data. We identified three patterns of data: (1) participants with complete outcome data at baseline and 12-month follow-up (n=52); (2) participants who were lost to follow-up during or immediately postintervention (n=21); and (3) participants who were lost to follow-up at the 1-, 3-, 6-, or 12-month follow-up time points (n=29). We used the conservative approach of assuming that participants belonging to pattern 2 did not benefit from the intervention, and we set the EMM at 6 and 12 months of follow-up equal to the EMM at baseline for these participants. For group 3, we estimated the EMM at the 6- and 12-month follow-ups as being equal to the EMMs across the first three time points of the intervention (baseline to week 4). The overall treatment effect at 6 or 12 months of follow-up was then defined as a linear combination of EMMs for the three different patterns, weighted by the proportion of participants belonging to each pattern. The standard error for this estimate was calculated using the delta method (via the “car” package in R [51]), and a test of the null hypothesis of no treatment effect was performed using a Wald statistic. A full description of the LMM-based PMM procedure is provided in a previous paper [52].

#### Effect Size Calculation

For comparisons with previous literature, we calculated effect size (Hedges *g*) immediately postintervention and at 6 and 12 months after the intervention as the difference in outcome scores from baseline divided by the pooled weighted SD (based on observed outcome scores only). For the PMM, effect size (Cohen *d*) was calculated as the estimated reduction in outcome score (from the LMM-based PMM) divided by the SD of observed outcome scores at baseline. We also calculated the percentage of participants who met the definition for clinically significant improvement in PHQ-9 symptoms postintervention and at the 12-month follow-up, defined as either a 5-point reduction in PHQ-9 score from baseline or a 50% reduction in PHQ-9 score combined with a postintervention PHQ-9 score<10 (for participants with baseline scores ≥10).

#### Predictors of Outcome Change

Lastly, we performed exploratory multiple regressions to test whether participant demographics and engagement metrics predicted outcome change from baseline to 6 and 12 months postintervention with regard to PHQ-9 scores and from baseline to 6 months postintervention with regard to GAD-7 scores. In each case, we excluded participants who did not have an available change score and modeled the following predictor variables: age, gender, baseline PHQ-9 or GAD-7 score, total active days, and total days with therapist contact. Since antidepressant status was only available for a subset of participants, we repeated each analysis including and excluding antidepressant status. In addition, we excluded meditation minutes from all models, as this was highly correlated with active days. Predictors associated with a *P* value<.05 were considered significant.

## Results

### Demographics and Participation

Participant demographics for the primary group of participants with 12-month PHQ-9 data are presented in Table 1. On an average, participants were 33 years of age, had baseline symptoms above the cutoff for major depression (mean PHQ-9 score=12.8), and were predominantly female. In addition, 80 participants were based in Finland, while 22 were based in the United States. Participants based in the United States had significantly less PHQ-9 symptoms at baseline than those in Finland (United States: mean 8.33, SD 4.40; Finland: mean 13.9, SD 4.82; *t*_101_=4.82; *P*<.001). Approximately one-third of the participants were taking antidepressant medication at the start of the intervention, and 20 participants (19.6%) dropped out of the intervention, where dropout was defined as less than 4 weeks of active participation during the 8-week intervention combined with incomplete PHQ-9 (depression) scores immediately postintervention.

**Table 1:** Participant demographics and relevant baseline data for the primary study cohort (with Patient Health Questionnaire-9 outcomes available up to 12-months postintervention).

Demographics and baseline data		All participants	Completed 8-week outcomes	Completed 6-month outcomes	Completed 12-month outcomes	Did not complete 8-week outcomes	Did not complete 6-month outcomes	Did not complete 12-month outcomes
Total participants, n (%)		102 (100)	83 (81.4)	44 (43.1)	52 (51)	19 (18.6)	58 (56.9)	50 (49)
Age in years, mean (SD)		32.9 (10.3)	33.2 (10.4)	33.5 (11.0)	31.3 (11.0)	31.9 (10.0)	32.5 (9.9)	34.6 (9.4)
**Gender, n (%)**								
	Male	23 (22.5)	19 (22.9)	11 (25)	10 (19.2)	4 (21.0)	12 (20.7)	13 (26)
	Female	79 (77.5)	64 (77.1)	33 (75)	42 (80.8)	15 (79.0)	46 (79.3)	37 (74)
**Antidepressants, n (%)**								
	Yes	25 (24.5)	18 (21.7)	7 (15.9)	7 (13.5)	7 (36.8)	18 (31.0)	18 (36)
	No	44 (43.1)	37(44.6)	24 (54.6)	22 (42.3)	7 (36.8)	20 (34.5)	22 (44)
	Unknown	33 (32.4)	28 (33.7)	13 (29.6)	23 (44.2)	5 (26.3)	20 (34.5)	10 (20)
**Country, n (%)**								
	Finland	80 (78.4)	67 (80.7)	36 (81.8)	48 (92.0)	13 (68.0)	44 (75.9)	32 (64.0)
	Unites States	22 (21.6)	16 (19.3)	8 (18.2)	4 (8.0)	6 (32.0)	14 (24.1)	18 (36.0)
Baseline PHQ-9^a^ score, mean (SD)^b^		12.8 (5.2)	13.0 (5.4)	13.2 (5.8)	14.3 (5.0)	11.6 (4.6)	12.5 (4.8)	11.2 (5.0)

^a^PHQ-9: Patient Health Questionnaire-9.

^b^Significantly associated with the presence of PHQ-9 scores at the 12-month follow-up.

Just over half (51%) of all participants reported a PHQ-9 outcome at 12 months postintervention (Table 1). Logistic regression revealed that participants with higher depression scores at baseline (*B*=–0.11, *P*=.02) and those who engaged with the intervention on more days (*B*=–0.05, *P*=.009) were more likely to complete the PHQ-9 at 12 months postintervention. When including country as a covariate, participants in the United States were less likely to complete PHQ-9 data at 12 months postintervention than participants in Finland (*B*=1.89, *P*=.01), although this may have been driven by differences in baseline PHQ-9 severity.

We also report participant demographics for a separate group of patients with anxiety outcomes available at the 6-month follow-up in Table 2. On an average, baseline GAD-7 scores suggest that participants had symptoms above the cutoff for moderate anxiety (mean GAD-7 score=10.7), despite being recruited on the basis of symptoms of depression. Age, gender, country, and baseline PHQ-9 symptoms were similar to those of the primary study cohort presented in Table 1. Intervention engagement positively predicted completion of the GAD-7 scale at 6 months postintervention (*B*=–0.07, *P*<.001), but baseline GAD-7 scores did not (*P*=.99).

**Table 2:** Participant demographics and relevant baseline data for patients with Generalized Anxiety Disorder-7 outcomes available at 6-months postintervention.

Demographics and baseline data		All participants	Completed 8-week outcomes	Completed 6-month outcomes	Did not complete 8-week outcomes	Did not complete 6-month outcomes
Total participants, n (%)		102 (100)	74 (72.6)	45 (44.1)	28 (27.5)	57 (55.9)
Age in years, mean (SD)		31.7 (11.4)	31.5 (11.7)	31.0 (12.0)	32.1 (10.8)	32.2 (10.9)
**Gender**, n (%)						
	Male	16 (15.7)	9 (12.2)	4 (8.9)	7 (25.0)	12 (21.1)
	Female	86 (84.3)	65 (87.8)	41 (91.9)	21 (75.0)	45 (78.9)
**Antidepressants**, n (%)						
	Yes	47 (46.1)	37 (50.0)	22 (48.9)	10 (35.7)	25 (43.9)
	No	53 (52.0)	36 (48.7)	23 (51.1)	17 (60.1)	30 (52.6)
	Unknown	2 (1.9)	1 (1.4)	0 (0)	1 (3.6)	2 (3.5)
**Country**, n (%)						
	Finland	90 (88.2)	71 (95.9)	45 (100)	19 (67.9)	12 (21.1)
	United States	12 (11.8)	3 (4.1)	0 (0)	9 (32.1)	45 (78.9)
Baseline GAD-7^a^ score, mean (SD)		10.7 (4.9)	11.0 (4.8)	10.8 (4.8)	9.85 (4.9)	10.5 (4.9)
Baseline PHQ-9^b^ score, mean (SD)		13.4 (4.9)	13.6 (4.7)	13.1 (4.4)	12.9 (5.5)	13.7 (5.4)

^a^GAD-7: Generalized Anxiety Disorder-7.

^b^PHQ-9: Patient Health Questionnaire-9.

### Engagement

Week-by-week intervention engagement rates are summarized in Table 3. On an average, participants engaged with the intervention on 31.3 days (SD 13.5, min=3, max=56), which corresponds to 55.9% of intervention days (where engagement is defined as >3 minutes of app-based mindfulness meditation on a given day). Participants completed an average of 9.79 hours of mindfulness-based exercises (SD 5.01, min=0.79, max=24.7) and had contact with their therapist on 13.1 days (SD 8.34, min=0, max=35) or 23.4% of intervention days. In addition, 68 participants (66.7%) completed at least one app-based meditation practice on each of the 8 weeks of the intervention. As reported in a previous publication [34], the mean number of days of intervention engagement (*F*_7,707_=42.4, *P*<.001) and the mean number of days of therapist contact (*F*_7,707_=5.37, *P*<.001) decreased from week 1 to week 8 (Table 3). Of the 102 participants, 81.4% completed the PHQ-9 immediately postintervention, while 43.1% and 51% completed the PHQ-9 at 6 and 12 months postintervention, respectively.

**Table 3:** Week-by-week intervention engagement across all participants. “Active days” corresponds to >3 minutes of app-based mindfulness practice on a given day.

Metric	Week							
	1	2	3	4	5	6	7	8
Active days, mean (SD)	5.02 (1.81)	4.65 (1.89)	4.54 (1.95)	4.13 (1.95)	3.76 (2.19)	3.59 (2.34)	2.95 (2.21)	2.67 (2.20)
Meditation minutes, mean (SD)	93.9 (39.0)	105.9 (52.6)	80.6 (40.0)	76.1 (47.6)	82.6 (60.5)	67.7 (55.2)	39.0 (39.8)	41.5 (39.5)
Days with therapist contact, mean (SD)	2.02 (1.27)	1.90 (1.53)	1.65 (1.40)	1.75 (1.40)	1.47 (1.42)	1.46 (1.45)	1.43 (1.43)	1.42 (1.37)

### Patient-Reported Outcomes

#### Depression Symptoms

**Figure 1:**
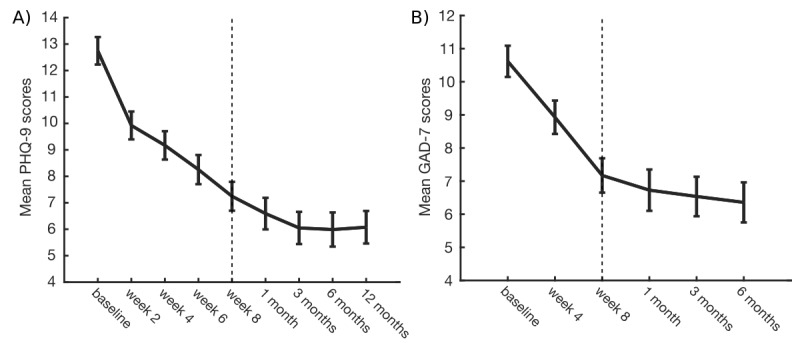
Estimated marginal means for PHQ-9 (A) and GAD-7 (B) scores across all available timepoints. The dotted line indicates the last week of the 8-week intervention. Error bars represent standard error of the mean. PHQ-9: Patient Health Questionnaire (9-item version); GAD-7: Generalized Anxiety Disorder (7-item version).

Participants reported clinically significant improvements in depression symptoms (PHQ-9 scores) from baseline to postintervention (5.50-point reduction, 95% CI 4.58-6.42; *P*<.001, Hedges *g*=1.02, 95% CI 0.71-1.32; Figure 1, panel A). This improvement was maintained at 6 months (6.76-point reduction, 95% CI 5.61-7.90; *P*<.001; Hedges *g*=1.30, 95% CI 0.91-1.68) and 12 months (6.67-point reduction; 95% CI 5.59-7.75; *P*<.001; Hedges *g*=1.14, 95% CI 0.78-1.49) postintervention. The improvement at 6 months remained robust when including an additional 95 participants (total n=197) with outcomes available at 6 months postintervention only (6.53-point reduction, 95% CI 5.73-7.33; *P*<.001; Hedges *g*=1.28, 95% CI 1.00-1.55).

When considering participants with PHQ-9 scores≥10 at baseline (n=83), 48% reported a clinically significant improvement immediately postintervention, which increased to 60% at the 12-month follow-up (defined as postintervention score<10 combined with ≥50% symptom reduction). When including all participants and using the more liberal definition of a 5-point reduction in PHQ-9 symptoms, 56% reported clinically significant improvements immediately postintervention, which increased to 77% at the 12-month follow-up.

#### Sensitivity Analysis

We also estimated PHQ-9 scores at 6 and 12 months postintervention under the conservative assumption that participants with missing data did not experience any long-term benefit from the intervention or benefitted only marginally (see Methods). This approach revealed more modest yet significant reductions in PHQ-9 scores relative to baseline at both 6 months (4.35-point reduction, 95% CI 3.65-5.06; *P*<.001; Cohen *d*=0.83, 95% CI 0.43-1.24) and 12 months (4.31-point reduction, 95% CI 3.63-4.99; *P*<.001; Cohen *d*=0.82, 95% CI 0.42-1.23) postintervention.

#### Anxiety Symptoms

Participants reported clinically significant improvements in anxiety (GAD-7 scores) from baseline to postintervention (3.45-point reduction, 95% CI 2.51-4.38; *P*<.001; Hedges *g*=0.69, 95% CI 0.38-1.00; Figure 1, panel B). This improvement was maintained at 6 months postintervention (4.26-point reduction, 95% CI 3.14-5.38; *P*<.001; Hedge *g*=0.91, 95% CI 0.54-1.28).

### Predictors of Outcome Change

Individuals with higher PHQ-9 symptoms (*b*=0.54, *P*<0.001) or higher GAD-7 symptoms (*b*=0.63, *P*<.001) at baseline were likely to experience larger reductions in depression (at the 12-month follow-up) and anxiety (at the 6-month follow-up), respectively. However, none of the reported engagement metrics or participant demographics were predictive of score change from baseline to 6- and 12-month follow-ups, for either PHQ-9 or GAD-7 (all *P*>.05).

## Discussion

### Principal Findings

The Meru Health *Ascend* intervention is a newly developed, smartphone-based, therapist-supported intervention for depression and anxiety, designed to overcome common barriers to treatment. A recent feasibility study reported that the intervention is feasible and associated with reduced depression symptoms immediately after the intervention [34]. This follow-up study extends these findings by demonstrating that the intervention is associated with clinically significant reductions in symptoms of both depression and anxiety and that these reductions are maintained up to 1 year and 6 months after intervention completion, respectively.

Understanding whether improvements in symptoms associated with *Ascend* are maintained long-term is important, as evidence suggests that a large proportion of patients are likely to relapse following treatment for depression or anxiety [30,32]. Although relapse rates associated with other smartphone-based interventions for depression and anxiety are largely unknown, previous research suggests that the risk of relapse is substantially lower following psychotherapy than following pharmacotherapy, where as many as 50%-75% of patients are likely to experience significant return of symptoms within 12 months of withdrawal from the latter [53,54]. Our results suggest that, similar to in-person psychotherapy, *Ascend* may be associated with enduring effects that extend for up to 12 months beyond the end of treatment. However, further work is needed to understand the long-term effectiveness of *Ascend* and other smartphone-based interventions for depression and anxiety under controlled conditions and relative to conventional treatment methods.

Our analysis revealed a larger effect size for the reduction in depression symptoms 12 months postintervention (*g*=1.14) than was reported by a recent meta-analysis [26] comparing smartphone-based interventions for depression to inactive control groups (*g*=0.56). However, this study was uncontrolled, and we therefore caution the reader not to overinterpret this difference. Indeed, the large effect reported here is consistent with other within-group (uncontrolled) effect sizes for app- and online-based depression interventions [55-57]. Further, a recent meta-analysis reported a similar uncontrolled effect size of *g*=1.29 for the reduction in depression symptoms associated with transdiagnostic, internet-delivered cognitive-behavioral therapy for depression and anxiety at follow-up [33]. Together, this suggests that the aforementioned discrepancy may stem from the lack of a comparison group in our study. We observed that the reduction in depression symptoms remained significant when making a conservative assumption that participants with missing data did not experience any long-term benefit from the intervention. However, the average reduction in depression under this assumption was marginally below the threshold for clinical significance (5-point change in PHQ-9 symptoms).

Similarly, our study revealed a slightly higher effect size for the reduction in anxiety symptoms immediately after the intervention (*g*=0.69) and 6 months postintervention (*g*=0.91) than was recently reported in a meta-analysis [27] comparing smartphone-based interventions for anxiety symptoms to inactive or waitlist control groups (*g*=0.45) [27]. This discrepancy may also be explained by the fact that this study was uncontrolled, and the effect size reported here may thus be an overestimate. In addition, the interventions included in the aforementioned meta-analysis were approximately 2 weeks shorter (on average) than the current 8-week intervention. Further, this study recruited participants on the basis of elevated symptoms of depression (as opposed to anxiety), making it difficult to make direct comparisons. Interestingly, the slightly higher effect size reported here is consistent with the results from online (as opposed to app-based) psychological interventions for anxiety disorders [58,59] and is, in fact, slightly lower than the effect size reported by a recent meta-analysis [33] of transdiagnostic, internet-delivered cognitive-behavioral therapy for depression and anxiety (uncontrolled pretest to follow-up, *g*=1.29).

The finding that *Ascend* was associated with reductions in symptoms of anxiety as well as depression is unsurprising, given that the intervention comprises evidence-based practices that have previously been shown to be effective at treating both conditions [60]. In addition, depression and anxiety are highly comorbid, suggesting the presence of shared underlying mechanisms that may respond to similar treatment methods [61]. Indeed, baseline scores suggest that a large proportion of participants were experiencing anxiety symptoms before the intervention, despite being recruited on the basis of having symptoms of depression. However, the magnitude of symptom reduction associated with *Ascend* was slightly larger for depression than for anxiety, likely reflecting the fact that the intervention was designed to address depression as the primary condition. Although previous online psychological interventions have often separated the treatment of depression and anxiety symptoms, there has been a recent trend toward the development of transdiagnostic interventions that target mechanisms common to multiple psychiatric disorders. These results add to a growing body of evidence highlighting the feasibility of such interventions [33] and suggest that smartphone apps may have the potential to address symptoms of depression and anxiety concurrently.

Two important but largely unanswered questions are who is likely to benefit the most from app-based psychological interventions and what factors are predictive of the degree to which individuals benefit over time. Our previous feasibility study suggested that a greater volume of app-based practice predicted the occurrence of fewer depressive symptoms 4 weeks after completing the *Ascend* intervention [34]. In this study, the degree of engagement with *Ascend* did not impact depression or anxiety scores at 6 or 12 months postintervention, although this analysis was likely underpowered due to a large proportion of missing data at these time points. It is also likely that a portion of the variance in scores at these time points was driven by factors not captured by our study. Further research is needed to understand the factors and components of the intervention that are predictive of long-term benefits.

### Limitations

As with the former feasibility study [34], this study used a nonrandomized, uncontrolled design, which precludes causal inferences regarding the intervention and patient-reported outcome changes. In addition, the effect sizes reported in this study are likely an overestimate of the true treatment effect relative to an active control or treatment as usual.

Further, approximately one-third of the participants were taking antidepressants during (and presumably after) the intervention, making it difficult to preclude the possibility that the reduction in depression symptoms was caused or maintained by antidepressants [62] or by the tendency for a proportion of depressed patients to naturally recover over a 12-month period [63] and not by this intervention. Future randomized controlled trials that compare *Ascend* to treatment as usual over an extended period are required to fully address these limitations. The Meru Health Online Clinic could also consider collecting information on the antidepressant status at follow-up (and not just at baseline) to better tease apart the long-term effects associated with the current intervention versus antidepressants.

Further, although engagement with the intervention was high, approximately half of all participants did not complete questionnaires at 6 and 12 months postintervention. Thus, our estimates of the percentage of participants that experienced clinically significant improvements in depression symptoms was based on the participants with complete data only. Further, participants with fewer symptoms of depression postintervention may have been more likely to engage with questionnaires, biasing estimates of the long-term treatment effect. This was addressed by using a robust pattern-mixture model approach and applying the most conservative clinical assumptions about the treatment effect for participants who dropped out or who were lost to follow-up. Nevertheless, such assumptions are inherently unverifiable, and recent guidelines highlight the importance of minimizing the likelihood of incomplete outcome data [64].

Although this study suggests that the intervention is associated with reductions in depression and anxiety symptoms, it does not address the underlying mechanisms or mediators of outcome change. Since the intervention encompasses techniques and practices from multiple approaches (cognitive behavioral therapy, mindfulness meditation, and behavioral activation therapy) as well as remote therapist and peer support, it is difficult to differentiate which components of the intervention are the most effective. Such information could assist with the design and optimization of future iterations of *Ascend* and other app-based interventions for mental health [65].

Finally, the study included self-selected participants who may have shown a higher degree of motivation to engage with the intervention. Further, the majority of participants were female, and although the study included individuals from both Finland and the United States, the majority of participants were based in Finland. Moreover, the Meru Health Clinic does not currently track patient race and ethnicity, making it difficult to assess the generalizability of the findings. Together, this suggests that this study sample may not be representative of the wider population of individuals with elevated symptoms of depression or anxiety, and future studies should address this issue by using more robust recruitment strategies and more representative study samples.

### Conclusions

Depression is a serious and growing problem that causes individual suffering and huge economic and societal costs worldwide. Many individuals with depression are unable to access appropriate treatment, with high costs and a lack of trained professionals being major barriers. Scalable, low-cost, app-based interventions such as *Ascend* are designed to overcome these barriers and may help to significantly reduce the burden of anxiety and depression. Further research is needed to investigate the efficacy of *Ascend* in comparison to control groups and other established treatments for depression and anxiety.

## Abbreviations

EMM: estimated marginal mean
GAD-7: Generalized Anxiety Disorder (7-item version)
HIPAA: Health Insurance Portability and Accountability Act
LMM: linear mixed effects model
PHQ-9: Patient Health Questionnaire (9-item version)
PMM: pattern mixture model
